# Development and psychometric validation of a novel patient survey to assess perceived quality of substance abuse treatment in South Africa

**DOI:** 10.1186/s13011-015-0040-3

**Published:** 2015-11-06

**Authors:** Bronwyn Myers, Rajen Govender, J. Randy Koch, Ron Manderscheid, Kim Johnson, Charles D. H. Parry

**Affiliations:** Alcohol, Tobacco and Other Drug Research Unit, South African Medical Research Council, Cape Town, South Africa; Department of Psychiatry and Mental Health, University of Cape Town, Cape Town, South Africa; Department of Sociology, University of Cape Town, Cape Town, South Africa; Department of Psychology, Virginia Commonwealth University, Richmond, VA USA; National Association of County Behavioral Health and Developmental Disability Directors, Washington DC, USA; Department of Psychiatry, Stellenbosch University, Cape Town, South Africa

**Keywords:** Patient survey, Patient-reported outcomes, Performance measurement, Service quality, Substance abuse treatment, South Africa

## Abstract

**Background:**

A hybrid performance measurement system that combines patient-reported outcome data with administrative data has been developed for South African substance abuse treatment services. This paper describes the development and psychometric validation of one component of this system, the South African Addiction Treatment Services Assessment (SAATSA).

**Methods:**

First, a national steering committee identified five domains and corresponding indicators on which treatment quality should be assessed. A decision was made to develop a patient survey to assess several of these indicators. A stakeholder work group sourced survey items and generated additional items where appropriate. The feasibility and face validity of these items were examined during cognitive response testing with 16 patients. This led to the elimination of several items. Next, we conducted an initial psychometric validation of the SAATSA with 364 patients from residential and outpatient services. Exploratory (EFA) and confirmatory factor analyses (CFA) were conducted to assess the latent structure of the SAATSA. Findings highlighted areas where the SAATSA required revision. Following revision, we conducted another psychometric validation with an additional sample of 285 patients. We used EFA and CFA to assess construct validity and we assessed reliability using Cronbach’s measure of internal consistency.

**Results:**

The final version of the SAATSA comprised 31 items (rated on a four-point response scale) that correspond to six scales. Four of these scales are patient-reported outcome measures (substance use, quality of life, social connectedness and HIV risk outcomes) that together assess the perceived effectiveness of treatment. The remaining two scales assess patients’ perceptions of access to and quality of care. The models for the final revised scales had good fit and the internal reliability of these scales was good to excellent, with Cronbach’s α ranging from 0.72 to 0.89.

**Conclusion:**

A lack of adequate measurement tools hampers efforts to improve the quality of substance abuse treatment. Our preliminary evidence suggests that the SAATSA, a novel patient survey that assesses patients’ perceptions of the outcomes *and* quality of substance abuse treatment, is a psychometrically robust tool that can help fill this void.

## Background

This paper describes the developmental process and measurement properties of a tool designed to assess South African patients’ perceptions of the quality and outcomes of substance abuse treatment. The prevalence of substance use disorders is high in South Africa where an estimated 13 and 6 % of the adult population meet DSM-IV criteria for a lifetime and past year diagnosis of a substance use disorder, respectively [1]. Untreated substance use disorders impact negatively on public health in South Africa through their association with risk for HIV and other infectious diseases [2], non-communicable diseases [3], and violence and injury [4]. Substance-related risks for HIV are of particular concern in South Africa where HIV prevalence is estimated to be 12.2 % among the general population [5].

Although South Africa has a fairly well-established substance abuse treatment system, questions have been raised about the quality and effectiveness of these services [6, 7]. Negative perceptions about the quality of substance abuse treatment are cause for concern as these perceptions often influence people’s decisions about whether or not to initiate treatment [6–8].

South Africa is one of many countries where there is concern about the quality of substance abuse treatment [9, 11]. In high-income countries, including the United States (US) [12, 13] and the United Kingdom (UK) [14, 15], these concerns have led to the development of performance measurement systems. These systems routinely collect data on a standardised set of indicators that reflect some aspect of treatment quality. When fully implemented, these systems are valuable as they generate information that can be used to monitor the quality of treatment, identify targets for quality improvement initiatives, and evaluate activities intended to improve performance [16, 17].

However, like most other low-and-middle income countries, South Africa lacks a system for monitoring the performance of its substance abuse treatment services [18, 19]. To fill this void, we set about developing such a system. During our formative work [19], South African substance abuse treatment providers reported willingness to implement a performance measurement system provided that it comprised brief, psychometrically robust measures that minimised burden for clinicians and administrators. Consequently, we developed a brief survey of patients’ (consumers’) perceptions of the quality and outcomes of treatment to be used, in conjunction with process measures constructed from treatment records, to routinely assess service quality.

Although treatment evaluation research has a long history of collecting objective outcomes data (such as [15, 20–22]), this is not the case in performance measurement systems routinely implemented in substance abuse treatment practices [16]. Many treatment providers are dissuaded from collecting outcomes data due to the costs associated with objectively assessing outcomes (either via biological testing or through using clinician-rated outcome measures) and concerns about the validity of self-reported substance use outcomes [16, 23]. As a result, most performance measurement systems rely on process indicators (such as indicators of treatment engagement and treatment completion) contained in administrative databases and treatment records to measure performance [11–13]. There are many advantages to using process data to assess quality of care. These data are relatively easy to collect and can directly identify specific areas of care that may require strengthening. There is also accumulating evidence that certain process indicators predict treatment outcomes [24–26]. For instance, US national treatment outcome studies have shown that longer stays in treatment are associated with better treatment outcomes at one and five year post-treatment [21, 22]. Despite these benefits, process indicators generally do not adequately reflect patients’ perceptions of care or their perceptions of how treatment affected their functional status and quality of life. Patient-reported outcome measures (PROMs), even though subject to the documented limitations of self-report data such as recall and response bias, can still reveal how patients experience and are responding to treatment [27]. In mental health settings, PROMs have been used to ensure that patients have a voice in treatment planning and service provision and to support the implementation of patient-centered care [28]. In addition, PROMs are feasible for under-resourced substance abuse treatment services to routinely collect as there are significantly fewer costs associated with collecting these measures compared with objective outcomes measures. Despite these advantages, surveys to assess PROMs are relatively novel in the substance abuse treatment field.

In our efforts to develop a performance measurement system for South African substance abuse treatment services, we took cognisance of the limitations of performance measurement systems based only on process data or reliant only on PROMs. To this end, we developed a hybrid system that combined patient-reported outcome data (collected using a patient survey) with process data from treatment records. In so doing, we wanted to provide an objective description of the treatment process while also accounting for patients’ experiences of and perceived responses to the treatment process. The aim of this article is to describe the development and measurement properties of one aspect of this system: the South African Addiction Treatment Services Assessment (SAATSA). Specific objectives were to: 1) describe the process of identifying domains and indicators on which to assess quality and outcomes; 2) describe the process of generating survey items for further assessment; 3) establish the feasibility of using this survey to assess treatment quality and outcomes and the face validity of survey items; and 4) examine the psychometric properties (construct validity and reliability) of the survey.

## Method

Guided by Streiner and Norman’s [29] framework for developing measurement scales, this study comprised five phases implemented from 2008 to 2013. The study received human subjects approval from the Centers for Disease Control and Prevention (CDC) and the University of Stellenbosch’s Health Research Ethics Committee (N10/03/105). All participants were asked to provide written informed consent for each phase of the study.

### Phase one: Selection of quality domains and indicators

A national steering committee, comprising key stakeholders and role players from the substance abuse treatment field, was formed to identify domains on which to assess treatment. This committee reviewed the domains used in other mental health and substance abuse performance measurement systems (e.g., [11–13]), and debated the relevance of these domains for South African services. The steering committee identified five domains on which treatment should be assessed: effectiveness, efficiency, access to treatment, person-centred services, and quality of services. Next, the committee generated a set of indicators (30 in total) that could be used to measure each domain. A Delphi consensus panel [30] comprising 36 content experts, was used to reduce the number of indicators. Of these stakeholders, 26 were substance abuse treatment providers, four were researchers and six were service planners. Each expert was asked to use a three-point scale (1 = low, 3 = high) to rate each indicator on how important each was an indicator of substance abuse treatment outcomes or quality of care, and feasibility of measurement. The research team calculated the mean importance, mean feasibility and a combined score for each indicator (using an importance: feasibility mean score ratio of 1:2) and ranked the indicators in order of their combined scores. The combined scores were used to reduce the number of indicators: those with a rating of 6.4 or higher (on a scale of 3 to 9) were retained, reducing the number of indicators to 18. After reducing the number of indicators, the steering committee decided whether to measure this indicator using a patient survey, data from treatment records or administrative data typically collected by treatment programmes. A description of the domains and corresponding indicators is provided in Table 1. This exercise revealed the need to develop a survey measuring patient-reported outcomes and perceptions of whether care was accessible, person-centred, and of acceptable quality.

**Table 1:** Summary of domains, indicators and methods of measurement for each indicator

Domain	Indicator	Mode of measurement		
		Patient Survey	Treatment records	Administrative data from services
Effectiveness	Decreased substance use	✓	✓	
	Increased/retained employment or return to/stay in school		✓	
	Increased retention in treatment		✓	✓
	Reduced sexual (HIV) risk behaviour	✓	✓	
	Improved quality of life	✓		
	Improved social connectedness	✓		
Efficiency	Length of delay in obtaining treatment		✓	✓
	Percentage of available treatment slots used			✓
Person-centered services	Proper assessment of client occurs (e.g., screening/assessment for mental illness and HIV/AIDS)			✓
	Client participation in the development and execution of the treatment plan	✓		✓
	Treatment is personalised to fit individual client’s needs	✓		
	Client received programme with linguistically and culturally appropriate content	✓		
Access	Length of time from request for services to first service	✓	✓	
	Affordability	✓		
	Equity- access to services is equitable for different age, race, and gender groups	✓	✓	
	Access to HIV testing and counselling and HIV risk reduction services	✓	✓	
Quality	Clients’ are engaged with the treatment programme	✓		
	Clients’ perceptions of services received are positive	✓		

### Phase two: Generation, rating and presentation of SAATSA items

From the steering committee, we developed a workgroup (comprising the research team and representatives from residential and outpatient treatment facilities) to generate a list of items to include in the patient survey. Based on a review of the literature, the survey workgroup identified the 18-item US Substance Abuse Perceptions of Care Survey [31, 32] as a tool that could be adapted for use in South Africa [31–33]. Where this survey did not address our indicators, committee members generated additional items. These additional items related to changes in sexual risk behavior, social connectedness, quality of life, and access to services. After generating several new items, the SQM steering committee made decisions about which items would be retained for the next stage of questionnaire development. This reduced the original set of 41 items to 33 items. Next, decisions were made about the instrument’s layout, the wording of instructions and items, and the response format. In this version of the SAATSA, all items were rated on a four point scale, with response options ranging from “strongly disagree” (1) to “strongly agree” (4). Each item also had a “not applicable” response option. Finally, we conducted a lexile analysis and revised the wording of items until every item was at a Grade 8 reading level.

### Phase three: Cognitive response testing

Next, the SAATSA was assessed for feasibility and face validity during cognitive response testing. Face validity refers to whether the instrument appears on face value to measure the key constructs it is purported to measure [33, 34]. The SAATSA was administered to 16 patients receiving substance use treatment between March and June 2011. Patients were recruited from two treatment sites (one residential and one outpatient) in KwaZulu-Natal (KZN) and two treatment sites (one residential and one outpatient) in the Western Cape (WC). The WC and KZN provinces were purposively selected for sampling because they serve diverse population groups with dissimilar patterns of drug use and have different types of treatment infrastructure. These two provinces, collectively, provide a good representation of the range of service providers and patients found nationally [19].

During cognitive response testing, patients were instructed to respond to each item as if they were completing the survey, and to identify items that were difficult to read or confusing. The length of time taken to complete the SAATSA was recorded. Patients were asked a series of questions about the clarity of the instructions and the items, what they understood they were being asked, suggestions for alternative wording of items, items that should be added or removed, and the overall format of the instrument.

### Phase four: Psychometric validation of the SAATSA

This phase explored the psychometric properties of the SAATSA scales (comprised of items developed in the previous phase) that were hypothesised to correspond to each of the quality domains. Objectives of this phase were to determine, by way of validity testing, the extent to which SAATSA items measured the relevant quality domains and to modify the SAATSA scales by eliminating items where necessary and justifiable.

### Sample, setting and procedure

We recruited a convenience sample of 364 patients from three treatment sites (one residential and two outpatient) in KZN (*n* = 134) and seven sites (three residential and four outpatient) in the WC (*n* = 230) between September 2011 and July 2012. This sample size is adequate for most factor analyses [35]. Participants were eligible to participate if they were at least 18 years of age, had been receiving substance abuse treatment for at least three weeks, and could read and write English. Treatment staff were asked to identify patients who met these inclusion criteria and assess their willingness to participate. Eligible participants were asked to provide written informed consent to participate in this pilot test.

Participants were predominantly male (76.1 %), of Coloured (mixed-race) ancestry (50.0 %) and their ages ranged from 18 to 74 years. Approximately 40 % of the sample had not completed high school. The most commonly reported primary substances of abuse were alcohol (39.8 %), followed by methamphetamine (34.3 %), heroin (7.1 %), and cannabis (6.5 %). Just over two-thirds (70.8 %) of the sample were receiving substance abuse treatment for the first time. Of the participants, 56.6 % were receiving residential treatment.

Participants self-completed the SAATSA and a brief demographic form in a private room at the site. These forms were placed in a sealed envelope and deposited in a secure box for safekeeping and collection by the research team.

#### Data analysis

The validation process used exploratory factor analysis (EFA) and confirmatory factor analysis (CFA) for validity testing. Listwise deletion of cases was used to address missing values, consequently all reported results are for cases with no missing values. Suitability of items for factor analysis was determined using two measures; Bartlett’s Test of Sphericity (significance of p ≤ 0.05) and the Kaiser-Meyer-Olkin (KMO) measure of sampling adequacy (0.70 and above). The EFA was conducted using maximum likelihood estimation (MLE) and oblique minimum rotation to extract and rotate identified factors [35]. Only factors with an Eigenvalue greater than 1 were selected for consideration [36]. The factor pattern matrix was used to examine loadings of items onto factors and to assess relevance and applicability of scale items to the latent construct, thereby directing decisions for item retention and removal.

Following this, CFA was used to confirm the latent structure observed in the EFA. The adequacy of the latent models was assessed using three critical fit indices [36]: χ^2^/df ratio (a ratio < 4 indicates good fit and < 2 indicates very good fit); Bentler Comparative Fit Index (CFI), CFI ≥ 0.90 indicates a good fit and CFI ≥ 0.95 indicates very good fit; and Root Mean Square Error of Approximation (RMSEA), RMSEA ≤ 0.08 and ≤ 0.05 indicates good fit and very good fit, respectively [37]. Where applicable, the significance of improvements in latent models were tested using Chi-Square tests. All statistical analyses were conducted using IBM SPSS and AMOS Version 21.0 for Windows [38].

### Phase five: Construct validity and reliability testing

Findings from phase four indicated that some of the SAATSA scales required further development. Consequently, we repeated the steps described in phases two and three, adding additional items. Phase five involved administering the revised SAATSA to patients receiving substance use treatment to establish the construct validity and internal consistency (reliability) of the SAATSA subscales.

#### Sample, setting and procedure

Using the same eligibility and recruitment processes described in phase four, 285 participants were recruited from treatment sites in KZN and the WC provinces between February 2013 and June 2013. Eligible patients who were willing to participate were asked to provide written informed consent. Participants then self-completed the SAATSA in a private room at the treatment facility. This was linked to a treatment admission form containing demographic data (but no personal identifiers) through a unique patient number. Both forms were placed in a sealed envelope and deposited in a secure box for collection by the research team.

Similar to the phase four, participants were predominantly male (79.6 %), of Coloured ancestry (56.7 %) and their ages ranged from 18 to 71 years. Over two-thirds of the sample (70.7 %) had not completed high school. The most commonly reported primary substances of abuse were methamphetamine (38.0 %), followed by alcohol (34.5 %), heroin (8.7 %), and cannabis (8.4 %). Most participants (70.1 %) were receiving substance abuse treatment for the first time. Of the participants, 53.3 % were receiving residential treatment.

#### Data analysis

The techniques and analysis parameters discussed in phase four were applied to examine the latent structure of the revised scales. In addition, after the scales had been finalised, scale reliability was assessed using Cronbach’s measure of internal consistency. A Cronbach’s alpha coefficient of at least 0.70 is considered acceptable, while ≥0.80 is desirable [29]. In all analyses, listwise deletion of cases was used to address missing values, thus reported results are limited to cases with no missing values. All statistical analyses were conducted using IBM SPSS and AMOS Version 21.0 for Windows [38].

## Results

### Results of cognitive response testing

Most participants reported that the SAATSA was easy to complete (94 %) and understand (94 %). All participants reported that they could complete the SAATSA with very little assistance. Based on participants’ responses to the items, several changes were made to the SAATSA: seven items were discarded and five items were revised and reworded for clarity or comprehension (see Table 2). These changes shortened the SAATSA to 26 items. These 26 items were thought to constitute six scales: three patient-reported outcome measures (PROMs) assessing treatment effectiveness (specifically changes in substance use, quality of life and social connectedness), and scales assessing perceptions of the accessibility, person-centredness and quality of care.

**Table 2:** Changes made to the SAATSA as a result of cognitive response testing

Items for cognitive response testing	Changes made to item as a result of testing
1a. The amount of time between when I asked for and received help was OK.	Discard
1b. The amount of time I had to wait to get services was acceptable to me.	Retain
2a. It is difficult to pay for my treatment.	Discard
2b. I can afford my treatment.	Revise: I can afford the treatment I want to receive
3. The staff at this treatment centre are sensitive to my culture.	Replace: The staff at this treatment centre treat me with respect
4. The staff at this treatment centre are sensitive to my gender.	Discard
5. The people I went to for treatment services spent enough time with me.	Revise: The people I went to for treatment services at this centre spent enough time with me.
6. I help to plan my treatment.	Revise: I have a say in deciding about my substance abuse treatment that I am receiving here
7a. I have received enough information about the different services.	Discard
7b. The staff at this treatment centre told me about other services in the community.	Revise: The staff at this treatment centre told me about services in my area that will help me stay off drugs and alcohol
8a. I have access to all the services I need in this treatment centre for my recovery	Discard
8b. I am given a choice of services in this treatment centre.	Retain
9a. This treatment centre teaches me how to avoid getting HIV.	Retain
9b. This treatment centre helps me reduce my HIV risk.	Discard
10. The staff at this treatment centre are sensitive to my background.	Retain
11. My general health is improving.	Retain
12. I am better able to cope when things go wrong.	Retain
13. I am better able to accomplish the things I want to do.	Retain
14. I am less likely to use alcohol or other drugs.	Retain
15. I am doing better at work/finding work or at school.	Retain
16. I am more likely to practice safe sex.	Retain
17. There is someone who cares about whether I am doing better.	Retain
18. I have someone who will help me when I have a problem.	Retain
19. I have people in my life who are a positive influence.	Retain
20. The people who care about me are supportive of my treatment.	Retain
21. My friends and family are more able to count on me.	Retain
22. I have friends who are not using alcohol or drugs.	Retain
23. I have someone who will listen to me when I need to talk.	Retain
24a. I think the use of alcohol and drugs is a problem for me.	Discard
24b. I now know that using alcohol and drugs is a problem for me.	Retain
25. I need to work on my problems with alcohol and/or drugs.	Retain
26. The treatment centre is helping me to recover from using drugs and alcohol.	Retain
27. I would recommend this treatment centre to a friend	Retain

### Initial validation of the SAATSA scales

Results of the initial EFA generally supported the latent structure of the effectiveness domain. The 12 effectiveness items loaded onto three factors relating to substance use, quality of life and social connectedness. To confirm this latent structure, a CFA was conducted specifying a one, two and three factor solution. The latter provided the best fit to the data, with all model indices having acceptable values (*χ*^2^/*df* ratio = 2.21; CFI = 0.98; RMSEA = 0.06, 90 % CI: 0.04, 0.09).

For items related to substance use, the EFA revealed a single factor that accounted for 52 % of the common variance. Three of the five items loaded reasonably well with one item loading poorly. CFA revealed that a trimmed four-item subscale provided a better fit for the data than the original five-item subscale, with all model indices being excellent (Table 3). For the five items thought to depict quality of life, a single factor emerged during EFA that accounted for 62 % of the common variance. Items 14 and 15 loaded poorly onto this factor. CFA revealed that a four-item version (excluding item 15) provided a better fit for the data than the original five-item version. For the seven items thought to reflect social connectedness, the EFA extracted a single factor accounting for 61 % of the common variance. Four items had high factor loadings, with a clear separation between these and the last three items. A CFA confirmed that a four-item version provided a better fit than a seven-item model (Table 3).

**Table 3:** Results of exploratory factor analyses (EFA) and confirmatory factor analyses (CFA) for the SAATSA substance use, quality of life and social connectedness subscales

SAATSA scales	EFA	CFA indices		
	Factor loading	*χ* ^2^/*df* ratio	CFI	RMSEA
Substance use scale				
13. I am less likely to use alcohol or other drugs	0.39	Five item version = 4.23. Four item version = 0.97	Five item version = 0.96. Four item version = 0.99	Five item version = 0.09 (CI: 0.07, 0.18) Four item version = 0.01 (CI: 0.00, 0.04)
21. I have friends who are not using alcohol or drugs	0.21			
23. I know that using alcohol or drugs is a problem for me	0.50			
24. I need to work on my problems with alcohol and/or drugs	0.33			
25. The treatment centre is helping me recover from using drugs and alcohol	0.44			
Quality of life scale				
10. My general health is improving	0.48	Five item version = 3.56. Four item version = 2.75	Five item version = 0.98. Four item version = 0.99	Five item version = 0.08 (CI: 0.06, 0.15). Four item version = 0.07 (CI: 0.02, 0.10)
11. I am better able to cope when things go wrong	0.45			
12. I am better able to accomplish the things I want to do	0.56			
14. I expect to do better at work/finding work or at school	0.41			
15. I am more likely to practice safe sex	0.32			
Social connectedness scale				
16. There is someone who cares about whether I am doing better	0.61	Seven item version = 2.86. Four item version = 2.25.	Seven item version = 0.98. Four item version = 0.99.	Seven item version = 0.07 (CI: 0.01, 0.12). Four item version = 0.06 (CI: 0.02, 0.09).
17. I have someone who will help me when I have a problem	0.66			
18. I have people in my life who are a positive influence	0.64			
19. The people who care about me are supportive of my treatment	0.68			
20. My friends and family are more able to count on me	0.51			
21. I have friends who are not using alcohol or drugs	0.26			
22. I have someone who will listen to me when I need to talk	0.49			

The EFA revealed that further developmental work was required for the access and quality of care domains. There were only two items referring to access, one of which had a large amount of missing data. For this reason it was removed from the analysis, rendering the single-item scale invalid. Similarly, two of the three items initially thought to belong to the person-centredness domain seemed to reflect quality of care; the removal of these items rendered this scale invalid. Consequently, the one remaining access item together with the three items on person-centred care and five items on perceived quality of services were subsumed into a nine-item factor thought to refer to overall quality of care. The EFA on this scale indicated two factors that accounted for 53 % of the common variance, suggesting that not all items were working together consistently. Item 7 was the most problematic. A CFA found that a nine-item single factor solution provided a poor fit to the data, with all model indices having unacceptable values (*χ*^2^/*df* ratio = 5.04; CFI = 0.86; RMSEA = 0.10, 90 % CI: 0.06, 0.16) whereas model indices for an eight-item solution (excluding item 7) were better (*χ*^2^/*df* ratio = 1.84; CFI = 0.98; RMSEA = 0.05, 90 % CI: 0.02, 0.09). However, as some items were still not loading well, we decided to reformulate these to remove conceptual ambiguities. As access to treatment is a domain in our quality framework, we also decided to develop additional items related to service access. In addition, we decided to develop another outcome measure, the HIV risk behaviour subscale, to better assess changes in HIV risk behaviour as a result of treatment.

In addition, there were a number of missing responses largely due to the presence of a “not applicable” response category for each of the SAATSA items (35 % of cases had at least one item for which there was a missing response). To reduce the number of missing responses, we decided to remove the “not applicable” option from most of the SAATSA items, except in those instances where it was conceptually relevant (e.g., items measuring sexual behaviour for individuals not engaging in sexual activity). Given these revisions to the SAATSA items and response categories, we conducted a further pilot study to assess the psychometric properties of the revised 39-item SAATSA.

### Construct validity and reliability of SAATSA scales

Changes to the SAATSA response categories reduced the number of missing responses and significantly improved the factor loadings of the SAATSA items. In this study, less than 15 % of cases had missing responses, as compared to 35 % in the first pilot study. Findings for the items reflecting change in substance use generally confirmed the results of phase four. A single factor emerged with the item “I have friends who are not using alcohol or drugs” loading poorly onto the factor (Table 4). CFA revealed that a four-item version (excluding this item) provided a much better fit for the data than a five-item solution. Consequently we discarded this item. This four-item scale demonstrated good internal consistency (Cronbach’s α = 0.72).

**Table 4:** Construct validity and reliability of the revised SAATSA subscales

SAATSA scales	EFA	CFA indices			Internal consistency
	Factor loading	*χ* ^2^/*df* ratio	CFI	RMSEA	Cronbach’s α
Substance use scale					
17. I am less likely to use alcohol or other drugs	0.44	Five item version = 1.82. Four item version = 0.59	Five item version = 0.99. Four item version = 0.99	Five item version = 0.05 (CI: 0.00, 0.10) Four item version = 0.01 (CI: 0.00, 0.07)	Cronbach’s α (4 item scale) = 0.72
25. I have friends who are not using alcohol or drugs	0.30				
27. I know that using alcohol or drugs is a problem for me	0.83				
28. I need to work on my problems with alcohol and/or drugs	0.62				
29. The treatment centre is helping me to recover from using drugs and alcohol	0.74				
Quality of life scale					
14. My general health is improving	0.66	Five item version = 5.18. Four item version = 3.52	Five item version = 0.92. Four item version = 0.99	Five item version = 0.11 (CI: 0.07, 0.19). Four item version = 0.08 (CI: 0.04, 0.15).	Cronbach’s α (4 item scale) = 0.81
15. I am better able to cope when things go wrong	0.77				
16. I am better able to accomplish the things I want to do	0.72				
18. In the future I will be more likely to do better at work or at school	0.40				
19. I am more likely to practice safe sex	0.59				
Social connectedness scale					
20. There is someone who cares about whether I am doing better	0.79	Seven item version = 1.84. Six item version = 1.61	Seven item version = 0.98. Six item version = 0.99	Seven item version = 0.06. (CI: 0.02, 0.12). Six item version = 0.05 (CI: 0.00, 0.09).	Cronbach’s α (6 item scale) = 0.89
21. I have someone who will help me when I have a problem	0.84				
22. I have people in my life who are a positive influence	0.76				
23. The people who care about me are supportive of my treatment	0.74				
24. My friends and family are more able to count on me	0.60				
25. I have friends who are not using alcohol or drugs	0.36				
26. I have someone who will listen to me when I need to talk	0.65				
HIV risk scale					
33. I am more likely to use condoms when I have sex.	0.69	Single scale = 3.27	Single scale = 0.94	Single scale = 0.07 (CI: 0.03, 0.12).	Cronbach’s α = 0.81
34. I am less likely to use alcohol and drugs before I have sex.	0.45	Two scales =1.68	Two scales =0.98	Two scales =0.04 (CI: 0.01, 0.09).	
35. I am more likely to only have sex with one partner.	0.32				
36. I am more likely to use condoms properly.	0.71				
37. I know it’s important to know my HIV status.	0.74				
38. I know where I can get tested for HIV.	0.77				
39. I know where I can get treatment for HIV.	0.78				
Quality scale					
8. The staff at this treatment centre treat me with respect.	0.56	Seven item version = 1.51. Six item version = 0.39	Seven item version = 0.98. Six item version = 0.99	Seven item version = 0.04. (CI: 0.00, 0.08). Six item version = 0.02 (CI: 0.00, 0.05).	Cronbach’s α (6 item scale) = 0.74
9. The people I went to for treatment services spent enough time with me.	0.56				
10. I have a say in deciding about my substance abuse treatment that I am receiving here	0.56				
11. The staff told me about services that will help me staff off alcohol and drugs.	0.46				
12. This treatment centre teaches me how to avoid getting HIV.	0.61				
13. The staff at this treatment centre are sensitive to my background	0.57				
30. I would recommend this treatment centre to a friend	0.62				
Access scale					
1. The amount of time I had to wait to get services was acceptable to me.	0.67	Six item version = 3.35. Five item version = 1.60	Six item version = 0.95. Five item version = 0.99	Six item version = 0.09. (CI: 0.06, 0.13). Five item version = 0.05 (CI: 0.00, 0.10).	Cronbach’s α (5 item scale) = 0.76
3. The location of this treatment centre is convenient for me.	0.66				
4. I feel safe travelling to this treatment centre.	0.69				
5. It is easy for me to obtain the treatments offered by this centre.	0.71				
6. My family is able to access services provided by this treatment centre.	0.59				
7. I can afford the transport costs of getting to this treatment centre	0.63				

For the five items relating to quality of life, a single factor emerged during EFA that accounted for 54 % of the common variance. The item “I will be more likely to do better at work or school” still loaded poorly onto this factor. CFA revealed that a four-item scale excluding this item provided a better fit to the data than a five-item scale. We therefore removed this item. This four-item scale demonstrated excellent internal consistency (Cronbach’s α = 0.81; Table 4).

Findings for the seven items relating to social connectedness also confirmed the results of phase four with a single factor emerging from the EFA. The item “I have friends who are not using alcohol or drugs” still loaded poorly onto the factor (Table 4). A CFA confirmed that a six-item version of the scale provided a better fit than the seven-item version; leading to the deletion of this item. The final scale demonstrated excellent internal consistency (Cronbach’s α = 0.89).

In this pilot iteration, we developed seven items relating to HIV. During EFA, all of the items loaded adequately onto a single factor accounting for 43 % of the common variance. Further inspection of the items suggested the possibility of a two factor solution, with some items referring to change in HIV risk behaviour and others to HIV knowledge. CFA revealed that a two factor model performed better than a single factor approach. However, as the data still supported the use of the measure as a single scale we decided to adopt this approach with the understanding that future applications could elect to apply the scale as a single or two factor solution. The internal consistency of this scale was excellent (Cronbach’s α = 0.81).

We had also expanded the access items from two to seven items. However, we excluded item 2, “I can afford the treatment I want to receive”, from the SAATSA as more than 40 % of the sample indicated that this was not applicable to them as they were receiving free services. For the six remaining items, a single factor emerged during EFA that accounted for more than 40 % of the common variance. Only one item referring to access by family members loaded poorly onto the factor (Table 4). CFA revealed that a five-item model (excluding this item) provided a better fit to the data than a six-item version. This item was removed from the SAATSA. The final five-item scale demonstrated good internal consistency (Cronbach’s α = 0.76).

For items related to perceived quality of services, EFA suggested a two factor solution that accounted for 42 % of the common variance. Only two items loaded onto the second factor: one loaded more strongly onto the first factor and the second (“The treatment centre teaches me how to avoid getting HIV”) loaded almost equally on both factors. A CFA was conducted to test whether or not removing this item from the subscale would result in better performance. Results from the CFA indicate that a six-item model excluding this item provided a better fit to the data than a seven-item solution (Table 4). We removed this item from the SAATSA. The final six-item scale demonstrated good internal consistency (Cronbach’s α = 0.76).

Overall, the results of the CFA led to the reduction of the SAATSA from 39 to 31 items (see Table 5 for final list of items). This reduction was desirable for scientific parsimony and because of the limited time available in treatment services for patient assessments.

**Table 5:** Final SAATSA scales and items

SAATSA scales	Scale items
Quality domain	
Access	Item 1. The amount of time I had to wait to get services was acceptable to me.
	Item 3. The location of this treatment centre is convenient for me.
	Item 4. I feel safe travelling to this treatment centre.
	Item 5. It is easy for me to obtain the treatments offered by this centre.
	Item 7. I can afford the transport costs of getting to this treatment centre
Quality	Item 8. The staff at this treatment centre treat me with respect.
	Item 9. The people I went to for treatment services spent enough time with me.
	Item 10. I have a say in deciding about substance abuse treatment I am receiving here.
	Item 11. The staff told me about services that will help me stay off drugs and alcohol.
	Item 13. The staff at this treatment centre are sensitive to my background.
	Item 30. I would recommend this treatment centre to a friend.
Effectiveness domain	
Quality of life	Item 14. My general health is improving.
	Item 15. I am better able to cope when things go wrong.
	Item 16. I am better able to accomplish the things I want to do.
	Item 19. I am more likely to practice safe sex.
Social connectedness	Item 20. There is someone who cares about whether I am doing better.
	Item 21. I have someone who will help me when I have a problem.
	Item 22. I have people in my life who are a positive influence.
	Item 23. The people who care about me are supportive of my treatment.
	Item 24. My friends and family are able to count on me more
	Item 26. I have someone who will listen to me when I need to talk.
Substance abuse	Item 17. I am less likely to use alcohol or other drugs.
	Item 27. I now know that using alcohol and drugs is a problem for me.
	Item 28. I need to work on my problems with alcohol and/or drugs.
	Item 29. The treatment centre is helping me to recover from using drugs and alcohol.
HIV	Item 33. I am more likely to use condoms when I have sex
	Item 34. I am less likely to use alcohol and drugs before I have sex
	Item 36. I am more likely to only have sex with one partner
	Item 37. I am more likely to use condoms properly
	Item 38. I know it is important to know my HIV status
	Item 39. I know where I can get tested for HIV
	Item 40. I know where I can get treatment for HIV

## Discussion

A lack of adequate measurement tools hampers efforts to improve the quality of substance abuse treatment. To help fill this void, we describe the development and measurement properties of a novel instrument, the SAATSA. To the best of our knowledge, this is the first tool designed to routinely assess patients’ perceptions of the outcomes *and* quality of substance abuse treatment. Although national treatment evaluation studies conducted in the US and elsewhere have collected both process and outcomes data, these data have been collected as part of large, often once-off research initiatives in which patients were tracked during and post-treatment (see for example [20–22, 39–41]). The SQM initiative differs from traditional treatment evaluation research in that it is a pragmatic performance measurement system designed to be continuously implemented by treatment providers (rather than researchers) for the purposes of routinely monitoring the quality of the services that they provide. Through a participatory and consensus-driven process, treatment stakeholders identified a set of domains and corresponding indicators for assessing substance abuse treatment quality. Next we created questionnaire items to measure indicators for these domains. The face validity of the questionnaire was established through review by experts, and through cognitive response testing with service users. The construct validity and initial reliability of the SAATSA was established during two rounds of pilot-testing with service users.

The rigorous manner in which the SAATSA was developed not only ensured a parsimonious representation of the underlying quality framework (by highlighting several items that could be eliminated), but also pointed to ways in which the SAATSA’s representation of the underlying quality of care framework could be improved. More specifically, we expected that factors identified during EFA would be related to four of the domains in our quality framework. We found support for our hypothesised effectiveness domain, with initial EFA and CFA demonstrating that this domain comprised three dimensions that corresponded to substance use, social connectedness and quality of life. However, these initial analyses did not provide support for separate domains pertaining to access to care, person-centred services and quality of care. We used these findings to guide the development of additional survey items relating to access to treatment as well as HIV risk behaviour.

With these revisions, the construct validity of the SAATSA improved, revealing better correspondence with our quality framework. More specifically, this next phase of EFA and CFA revealed four unique dimensions relating to the effectiveness domain: substance use, quality of life, social connectedness, and HIV risk. In addition, two dimensions were identified that relate to perceptions of access to treatment and quality of care. Additional analysis provided strong support for the reliability of these SAATSA scales. Taken together, these findings provide preliminary evidence that the SAATSA is a psychometrically sound measure of patient-reported quality and outcomes of substance abuse treatment.

The SAATSA can be grouped together with other types of measures described in the health services quality literature, in particular patient-reported outcome measures (PROMs) and tools for assessing perceptions of care. PROMs have been adopted as quality improvement tools in the UK [42], US [42, 43], Australia [44, 45] and Sweden [43]; although their use within substance abuse treatment services is limited. While some substance abuse services do use standardised tools to assess patient outcomes, these tools are clinician-administered (such as the Addiction Severity Index or Texas Christian University short forms [46]), and are either lengthy and time-consuming to use or they assess substance abuse symptom alleviation only. The SAATSA is distinct from these measures as it is a brief, self-administered tool that is able to assess substance abuse symptom reduction as well as patients’ perceptions of how treatment impacted on their quality of life and social connectedness- factors that are known to be important predictors of recovery [47]. In addition, unlike other standardised tools for measuring change in substance use, items contained in the SAATSA are anchored to the most recent treatment episode, making it suitable to use for managing performance of treatment services.

The SAATSA can also be grouped together with tools that assess perceptions of care and satisfaction with services such as Texas Christian University’s treatment satisfaction questionnaire [46]. However, the SAATSA is novel in that it examines patient-reported outcomes and perceptions of care in a single measure. Another strength of this tool is its brevity. On average, the SAATSA took less than 10 min to complete. Having one patient-administered tool that serves multiple functions and that poses little administrative burden for clinicians is likely to improve the chances of this tool being utilised in busy and under-resourced treatment settings [19].

While our focus has been on the development of a tool to assess the quality of South African substance abuse treatment services, we believe that the SAATSA can be applied more broadly. It was designed to be easily adaptable and can be modified to assess the quality of other levels of substance abuse intervention, although additional items specific to these services may need to be added to the tool. Second, items contained in the questionnaire are likely to be of relevance to treatment services in other similar low-and-middle income countries. However, the HIV risk scale may not be relevant in countries where HIV is not a significant health problem and in these countries, its use can be omitted.

The SAATSA was tested in a variety of treatment settings (outpatient/ambulatory, inpatient, residential), types and durations of treatment programmes. Although not reported here, we found no differences in the reliability of the subscales across different types of services. This improves confidence in its utility for the mix of treatment services available in other countries. However, more research is needed to understand the strengths and limitations of the questionnaire when it is used for other levels of substance abuse intervention and in other countries.

These findings should be considered in the light of some study limitations. First, we did not include former patients in the national steering committee, primarily because until very recently, substance abuse treatment service users in South Africa were not well organised into consumer interest groups. We acknowledge that treatment service users may have had different views of quality, however we believe we have mitigated this limitation through conducting considerable outreach to patients during the developmental phases of the SAATSA. In these phases, we were able to capture patients’ perceptions and understanding of treatment quality. Since these early developmental phases, the steering committee has been expanded to include former patients. Second, our sample was limited to patients who spoke English well and were at least 18 years of age. Although the SAATSA has since been translated into several other South African languages, further research is needed to establish the psychometric properties of these other language versions. Related to this, it is possible that some of the SAATSA items are not suitable for use with adolescent populations. We plan to use a similar process to develop an adolescent version of the SAATSA. Third, the methods used to validate the SAATSA were limited to factor analyses, although CFA was performed using robust analytic techniques. Additional testing is required to establish the predictive and convergent validity of the SAATSA scales. Related to this, the SAATSA is a self-report measure of patient outcomes and is subject to all of the well-documented limitations of self-report measures. Future research should consider comparing the performance of the SAATSA substance use scale against objective measures of drug use. Future research should also investigate the discriminant validity of the SAATSA scales – their ability to differentiate different treatment outcomes as well as subsamples of respondents. Further, the current examination of scale reliability was confined to tests of internal consistency, and it remains for future work to investigate the test-retest reliability of the SAATSA. Additionally, we did not have a sufficient sample size in either of these pilot studies to confirm our final model with a hold-out sample or to test for item invariance across multiple subgroups. Nevertheless, the fact that we found very similar findings for our analyses in two separate pilot tests is encouraging and suggestive of the stability of the SAATSA’s underlying factor structure.

## Conclusions

We have provided preliminary evidence supporting the construct validity and internal consistency of the SAATSA, suggesting that it is an appropriate tool for assessing the quality of substance abuse treatment. There are several ways in which this tool can be used. First, it can be used to identify themes around quality of care at specific practice settings that require further in-depth examination using qualitative approaches. Second, the SAATSA can be used as part of a participatory approach for identifying areas where programmes can be strengthened. In such an approach [48], providers are asked to provide inputs on their treatment setting which are used to understand the data generated from the SAATSA so that useful and practice-specific strategies for improving treatment quality can be developed. Third, it can be used as a monitoring tool to assess the impact of interventions to improve quality and outcomes of treatment. While findings suggest that the SAATSA is a psychometrically robust measure of patient-reported outcomes, it is only one component of a performance measurement system that seeks to combine patient-reported outcome data with administrative data. A remaining challenge is how to integrate these patient-reported outcomes with administrative data to ensure that this hybrid performance measurement model functions effectively. Ensuring this system functions properly is essential as the valid measurement of treatment quality could lead to improvements in treatment services, and ultimately better treatment outcomes.
